# The rs8506 TT Genotype in *lincRNA-NR_024015* Contributes to the Risk of Sepsis in a Southern Chinese Child Population

**DOI:** 10.3389/fpubh.2022.927527

**Published:** 2022-07-13

**Authors:** Jinqing Li, Huazhong Zhou, Bing Wei, Di Che, Yufen Xu, Lei Pi, Lanyan Fu, Jie Hong, Xiaoqiong Gu

**Affiliations:** ^1^Department of Clinical Biological Resource Bank, Guangzhou Women and Children's Medical Center, Guangzhou Medical University, Guangzhou, China; ^2^Department of Blood Transfusion, Guangzhou Women and Children's Medical Center, Guangzhou Medical University, Guangzhou, China; ^3^Pediatric Intensive Care Unit, Guangzhou Women and Children's Medical Center, Guangzhou Medical University, Guangzhou, China

**Keywords:** *lincRNA-NR_024015*, genetic polymorphisms, risk, children, sepsis

## Abstract

**Background:**

Sepsis is a highly life-threatening heterogeneous syndrome and a global health burden. Studies have shown that many genetic variants could influence the risk of sepsis. Long non-coding RNA *lincRNA-NR_024015* may participate in functional alteration of endothelial cell *via* vascular endothelial growth factor (VEGF) signaling, whereas its relevance between the *lincRNA-NR_024015* polymorphism and sepsis susceptibility is still unclear.

**Methods:**

474 sepsis patients and 678 healthy controls were enrolled from a southern Chinese child population in the present study. The polymorphism of rs8506 in *lincRNA-NR_024015* was determined using Taqman methodology.

**Results:**

Overall, a significant association was found between rs8506 polymorphism and the risk of sepsis disease (TT vs. CC/CT: adjusted OR = 1.751, 95%CI = 1.024–2.993, *P* = 0.0406). In the stratified analysis, the results suggested that the carriers of TT genotypes had a significantly increased sepsis risk among the children aged 12–60 months, females, early-stage sepsis and survivors (TT vs. CC/CT: OR_age_ = 2.413; OR_female_ = 2.868; OR_sepsis_ = 2.533; OR_survivor_ = 1.822; adjusted for age and gender, *P* < 0.05, respectively).

**Conclusion:**

Our study indicated that *lincRNA-NR_024015* rs8506 TT genotype might contribute to the risk of sepsis in a southern Chinese child population. Future research is required to elucidate the possible immunoregulatory mechanisms of this association and advance the development of novel biomarkers in sepsis.

## Introduction

Sepsis, a syndrome caused by a dysregulated immune response to infection, is a life-threatening worldwide health issue (1). Globally, there were an estimated 48.9 million cases of sepsis and 11.0 million potential sepsis-related deaths in 2017 (2). According to the age, sepsis incidence peaked in early childhood who were younger than 5 years, representing 41.5% (estimated 20.3 million) of overall cases of sepsis in 2017 (2). At present, despite advances in the diagnosis and treatment of sepsis, it remains a clinical challenge for clinicians and researchers due to the fact that sepsis is still the main cause of mortality worldwide (3). Hence, it is critical to investigate the potential biomarkers to evaluate the susceptibility and disease progression of sepsis.

Long non-coding RNA (lncRNA) is a type of RNA molecules with more than 200 nucleotides in length (4, 5). Although lacking of protein-coding capacity, lncRNA has been found to be related to so many pathological processes of human diseases, such as cancer and inflammatory response (4, 6). Recently, emerging studies have revealed that some lncRNAs, such as NEAT1, MALAT1, and ANRIL, could participate in the progression of sepsis and serve as a potential biomarker for sepsis (7–9). *LincRNA-NR_024015*, also known as Testis development related gene 1 (*TDRG1*), is a newly identified tumor-associated lncRNA. Studies have shown that lncRNA *TDRG1* could serve as a proto-oncogene in multiple tumor types (10–13). Chen and colleagues revealed that lncRNA *TDRG1* might promote endometrial carcinoma cell proliferation and invasion by targeting vascular endothelial growth factor (VEGF) (12). Severe vascular dysfunction is considered central to the progression of organ failure during the development of sepsis (14, 15). Furthermore, the elevated circulating level of VEGF was observed in patients with sepsis (16–20). According to these findings, we considered that *LincRNA-NR_024015* might have critical effects on the pathogenesis of sepsis by interacting with VEGF. In recent years, some studies have suggested that polymorphisms of the genetic components including inflammatory mediators and lncRNAs were markedly associated with the susceptibility of human sepsis (21–27). Nevertheless, whether the polymorphism of *lincRNA-NR_024015* can affect sepsis susceptibility is still unknown.

The present study aimed to investigate the association between the rs8506 polymorphism of *lincRNA-NR_024015* and sepsis susceptibility in the current hospital-based case–control study with 474 cases and 678 healthy controls in a southern Chinese child population.

## Materials and Methods

### Study Population

The study included 474 children with sepsis, who were randomly selected from January 2016 to December 2018 at Guangzhou Women and Children's Medical Center. The diagnostic criterial for sepsis, severe sepsis and septic shock were based on the Surviving Sepsis Campaign Guidelines for Management of Severe Sepsis and Septic Shock (28). The 678 subjects in the control group were recruited from the same hospital according to the age and gender of the children in an ~1:1 ratio. Sepsis patients and healthy controls who were older than 18 years and the patients with malignancy, immunodeficiencies, autoimmune, blood diseases or other organic diseases were excluded from the present study. The characteristics of all subjects in the present study were shown in Table 1. This study had received the ethics approval of the Institutional Review Board of Guangzhou Women and Children's Medical Center (2015042202). Written informed consent was obtained from each participant guardian.

**Table 1:** Frequency of selected characteristics in sepsis cases and healthy controls.

**Variables**	**Cases (*****n*** **=** **474)**		**Controls (*****n*** **=** **678)**		***P*-value^a^**
	**No**.	**%**	**No**.	**%**	
**Age (range, month)**	1–180	1–168	
Mean ± SD	23.78 ±35.16	25.93 ±30.81	0.272
<12	233	49.16	284	41.89	
12–60	194	40.93	323	68	
>60	47	9.92	71	10.47	
**Gender**					
Male	301	63.5	399	58.85	0.111
Female	173	36.5	279	41.15	
**Sepsis subtypes**					
Sepsis	99	20.89	–	–	–
Severe sepsis	290	61.18	–	–	–
Septic shock	85	17.93	–	–	–
**Prognosis**					
Survivors	394	83.12	–	–	–
Non-survivors	80	16.88	–	–	–
**Number of organs with dysfunction**					
1–2	276	58.23	–	–	–
3 or more	95	20.04	–	–	–
**Site of infection**					
Respiratory tract	296	62.45	–	–	–
Abdomen	28	5.91	–	–	–
Neurological	36	7.59	–	–	–
Bloodstream	35	7.38	–	–	–
Urinary tract	8	1.69	–	–	–
Other	71	14.98	–	–	–
**Type of infection**					
Gram-positive	241	50.84	–	–	–
Gram-negative	117	24.68	–	–	–
Polymicrobial	63	13.29	–	–	
Fungus	18	3.80	–	–	–
Negative	35	7.38	–	–	–

a *Two-sided χ^2^ test for differences between Sepsis patients cases and controls*.

*SD, standard deviation*.

### DNA Extraction and SNP Genotyping

The SNP with potential function and associated information were adopted according to the NCBI dbSNP database (http://www.ncbi.nlm.nih.gov/, http://snpinfo.niehs.nih.gov/) based on the following criteria: (1) the minor allele frequency (MAF) ≥5% in Southern Han Chinese offspring in HapMap; (2) affect the microRNA/Protein binding site activity; (3) located in the gene regulatory region. 2 ml of venous whole blood were collected in an EDTA tube from each sepsis child and control. Genomic DNA were extracted from the whole blood samples (200 μl) by the phenol–chloroform method using the TIANamp Blood DNA Kit (TianGen Biotech, Beijing, China) as described previously (29). The concentration and purity of the extracted DNA was determined by a NanoDrop 2000 (Thermo Fisher Scientific, USA). High-quality DNA samples with OD260/OD280 values between 1.6 and 1.8 were used for genotyping experiments. The allele-specific fluorescent probes (*lincRNA-NR_024015* rs8506) were purchased from Applied Biosystems (Thermo Fisher Scientific, USA). Genotyping for the rs8506 C>T was performed in the 384-well plate using Taqman PCR method (30). Briefly, DNA amplification was conducted in a volume of 5 μl containing 2.5 ng template DNA, primer pool, and 2 × Multiplex PCR Mix, followed by the conditions [95°C for 3 min, 40 cycles of (95°C for 20 s, 58°C for 90 s, 72°C for 30 s) and 72°C for 1 min] in an Applied Biosystems Q6 instrument (Thermo Fisher Scientific, USA). For quality control, three replicates for each sample were performed in a 384-well plate and each plate contained four distilled water samples instead of DNA templates. Moreover, 10% of the DNA samples was randomly selected for a repeated genotyping analysis, and the results were 100% consistent. Genotyping was performed blindly to the status of the case or control.

### Statistical Analysis

The differences in the genotype frequencies of *lincRNA-NR_024015* rs8506 and the demographic variables between sepsis cases and healthy controls were compared applying the χ^2^ test. Hardy–Weinberg equilibrium (HWE) of the healthy controls for rs8506 was tested using the goodness-of-fit χ^2^ test. Multivariate logistic regression analyses were carried out to calculate the odd ratios (ORs) and their 95% confidence intervals (CIs) for risk of sepsis, which were also stratified by the age, gender, prognosis and the number of organs with dysfunction. All of the statistical analyses were conducted using SAS software (Version 9.3; SAS Institute, Cary, NC, USA) and a *P*-value < 0.05 was considered to be statistically significant.

## Results

### Population Characteristics

In order to describe whether SNP in the *lincRNA-NR_024015* rs8506 was associated with susceptibility to sepsis in a southern Chinese child population, we carried out a case–control study with a cohort of 474 Chinese patients with sepsis and 678 healthy controls. The general and clinical characteristics of the study population were summarized in Table 1. On average, the patients were 24 months old (range: 1–180; standard deviation, SD: ±35) and the healthy controls were 26 months old (range: 1–168; SD: ±31). Of the 474 patients, 63% were male individuals and 37% were female; while 59% of the healthy controls were male and 41% were female. No statistically significant differences were found between cases and control groups with respect to age (*P* = 0.272) and gender (*P* = 0.111). During the observation period, 61.2% of the patients were in severe sepsis and 17.9% were in septic shock. Considering the prognosis of the patients, the patient group was subdivided into survivors (394; 83.1%) and non-survivors (80; 16.9%). Moreover, 58.2% of all the enrolled patients developed 1–2 organs with dysfunction; 3 or more organs with dysfunction occurred in about 20% of all the patients. Respiratory tract (62.45%), abdomen (5.91%), neurological (7.59%), bloodstream (7.38%) and urinary tract (1.69%) represented the predominant sites of infection in this study. Additionally, across all types of infection, the most frequent type was gram-positive infection (50.84%), followed by gram-negative infection (24.68%).

### Association Analysis

The genotypes of *lincRNA-NR_024015* rs8506 were successfully evaluated in the present study. As shown in Table 2, the distribution of the genotypes agreed with the Hardy–Weinberg equilibrium in the healthy controls (*P* = 0.234). We observed that the population who carried rs8506 TT genotype had a 1.755-fold higher risk of sepsis than those did not carry (TT vs. CC/CT: OR = 1.755, 95%CI = 1.028–2.996, *P* = 0.0394). Furthermore, after adjustments for age and gender, significantly elevated risk of sepsis was also found in the rs8506 TT genotype, as compared with rs8506 CC/CT genotype (TT vs. CC/CT: adjusted OR = 1.751, 95%CI = 1.024–2.993, *P* = 0.0406).

**Table 2:** Genotype frequency of *lincRNA-NR_024015* rs8506 in sepsis cases and healthy controls.

**Genotype**	**Cases (*N* = 474)**	**Controls (*N* = 678)**	***P*-value^a^**	**OR (95% CI)**	***P*-value**	**Adjusted OR (95% CI)^b^**	***P*-value^b^**
***lincRNA-NR_024015*** **rs8506 C>T (HWE** **=** **0.234)**						
CC	299 (63.08)	413 (60.91)	**0.0436**	1.000		1.000	
CT	144 (30.38)	239 (35.25)		0.832 (0.645–1.074)	0.1577	0.832 (0.645–1.075)	0.1592
TT	31 (6.54)	26 (3.83)		1.647 (0.958–2.832)	0.0712	1.644 (0.955–2.830)	0.0727
Dominant (CT+TT)	175 (36.92)	265 (39.09)	0.4563	0.912 (0.716–1.162)	0.4566	0.912 (0.716–1.163)	0.459
Recessive (CC+CT)	443 (93.46)	652 (96.17)	**0.039**	**1.755 (1.028–2.996)**	**0.0394**	**1.751 (1.024–2.993)**	**0.0406**

a *χ^2^ tests were used to determine differences in genotype distributions between the patients with sepsis and the healthy controls*.

b *Adjusted for age and gender. The values are shown in bold if P < 0.05*.

*CI, confidence interval; OR, odds ratio; HWE, Hardy–Weinberg equilibrium*.

### Stratified Analysis

We further performed a stratified analysis of the relationship between *lincRNA-NR_024015* rs8506 polymorphism and sepsis susceptibility by clinical features (Table 3). The rs8506 TT genotype was found to be markedly associated with an increased sepsis risk among the children aged 12–60 months (TT vs. CC/CT: OR = 2.377, 95%CI = 1.068–5.286, *P* = 0.0338; adjusted OR = 2.413, 95%CI = 1.083–5.376, *P* = 0.0311), females (TT vs. CC/CT: OR = 2.848, 95%CI = 1.218–6.658, *P* = 0.0157; adjusted OR = 2.868, 95%CI = 1.226–6.714, *P* = 0.0152), sepsis (TT vs. CC/CT: OR = 2.508, 95%CI = 1.139–5.522, *P* = 0.0224; adjusted OR = 2.533, 95%CI = 1.149–5.586, *P* = 0.0213), and survivors (TT vs. CC/CT: OR = 1.845, 95%CI = 1.061–3.209, *P* = 0.0301; adjusted OR = 1.822, 95%CI = 1.046–3.175, *P* = 0.0343). However, no significant associations were found in other stratified analyses.

**Table 3:** Stratification analysis of susceptibility in sepsis patients.

**Variables**	**CC+CT**	**TT**	***P*-value^a^**	**OR (95% CI)**	***P*-value**	**Adjusted OR (95% CI)^b^**	***P*-value^b^**
	**Patients/Controls**						
**Age, months**
<12	219/271	14/13	0.468	1.333 (0.614–2.894)	0.4681	1.327 (0.609–2.888)	0.4763
12–60	179/312	15/11	**0.0326**	**2.377 (1.068–5.286)**	**0.0338**	**2.413 (1.083–5.376)**	**0.0311**
>60	45/69	2/2	0.6757	1.533 (0.208–11.280)	0.6746	1.551 (0.210–11.431)	0.6669
**Gender**
Male	285/382	16/17	0.5161	1.262 (0.627–2.540)	0.5152	1.244 (0.617–2.508)	0.5411
Female	158/270	15/9	**0.0137**	**2.848 (1.218–6.658)**	**0.0157**	**2.868 (1.226–6.714)**	**0.0152**
**Sepsis subtypes**
Sepsis	90/652	9/26	**0.0334**	**2.508 (1.139–5.522)**	**0.0224**	**2.533 (1.149–5.586)**	**0.0213**
Severe sepsis	272/652	18/26	0.1142	1.660 (0.895–3.077)	0.1077	1.631 (0.878–3.032)	0.1218
Septic shock	81/652	4/26	0.7042	1.238 (0.422–3.638)	0.6973	1.240 (0.421–3.653)	0.6962
**Prognosis**
Survivors	367/652	27/26	**0.0309**	**1.845 (1.061–3.209)**	**0.0301**	**1.822 (1.046–3.175)**	**0.0343**
Non-survivors	76/652	4/26	0.6251	1.320 (0.449–3.884)	0.6138	1.334 (0.451–3.943)	0.6024
**Number of organs with dysfunction**, ***n*** **(%)**
1–2	260/652	16/26	0.1915	1.543 (0.815–2.924)	0.1832	1.499 (0.787–2.853)	0.2178
3 or more	89/652	6/26	0.2847	1.691 (0.677–4.221)	0.2607	1.720 (0.687–4.310)	0.2469

a *χ^2^ tests were used to determine differences in genotype distributions between the patients with sepsis and the healthy controls*.

b *Adjusted for age and gender. The values are shown in bold if P < 0.05*.

*CI, confidence interval; OR, odds ratio*.

## Discussion

In the present study, we enrolled a cohort of 474 cases with sepsis and 678 controls to evaluate the association between *lincRNA-NR_024015* rs8506 polymorphism and the sepsis susceptibility among southern Chinese children. The result showed that the carriers of rs8506 TT genotype had a significantly increased risk of sepsis when compared with that carrying CC/CT genotypes. Interestingly, the stratified analysis revealed that the increased risk level of rs8506 TT variant appeared more obvious in the children of 12–60 months old and female. Moreover, we also found that the rs8506 TT variant showed significantly elevated risk of sepsis in the subgroup of the patients who were in the early stage of sepsis or alive. Therefore, our findings provided evidences that *lincRNA-NR_024015* rs8506 TT genotype might be associated with the susceptibility of sepsis in a southern Chinese child population.

Long non-coding RNAs (lncRNAs) are a type of non-protein-coding RNAs which exceed 200 nucleotides in length (4, 5). It has been suggested that lncRNAs play important roles in the pathogenesis of various diseases through chromatin rearrangement, transcriptional control as well as post-transcriptional processing (4, 5). However, little is known about the relationship between lncRNAs and the sepsis susceptibility (7, 31). In the present study, we found that *lincRNA-NR_024015* rs8506 TT genotype was notably associated with an increased risk of sepsis. To our knowledge, this is the first study to evaluate the relationship of the rs8506 polymorphism with sepsis risk in a southern Chinese child population.

*lincRNA-NR_024015* (gene ID: 732253), namely *TDRG1*, was initially identified as a novel human testis-specific gene which served as a regulator in sperm motility and the development of testicular germ cell tumors (32, 33). Recent studies have suggested that *lincRNA-NR_024015* might play important roles in tumor progression in several cancer types including cervical (10), esophageal (34), ovarian (13) and endometrial carcinoma (12). Chen et al. (12) provided evidences that *lincRNA-NR_024015* might directly bound to VEGF-A protein and upregulated its expression, thus promoting the progression of endometrial carcinoma. Moreover, *lincRNA-NR_024015* and VEGF were found to be co-expressed and remarkably upregulated in fibrovascular membranes from diabetic retinopathy patients than those from epiretinal membrane (35). These reported data reveal that *lincRNA-NR_024015* might be beneficial to stimulate the VEGF pathway (35). VEGF is a potent mediator that not only increases the vascular permeability, but also promotes leukocytes adhesion by eliciting the expression of adhesive molecules (36–38). Actually, emerging data have suggested that circulating VEGF levels were elevated during the development of sepsis (16–20) and the levels of this factor were associated with sepsis severity and mortality (16–18). Furthermore, blockade of VEGF signaling in a mouse model might have beneficial effects on the survival of sepsis by decreasing inflammatory responses and endothelial permeability (39). Importantly, the expression level of *lincRNA-NR_024015* in esophageal tumor tissues with rs8506 CT and TT genotype was significantly higher than those with rs8506 CC genotype (34). Therefore, in combination with the findings in our study, we speculated that rs8506 TT genotype might increase the risk of sepsis *via* upregulating the levels of *lincRNA-NR_024015* and VEGF. Further study is needed to confirmed this possibility in the future.

Epidemiological studies have showed that global sepsis apparently occurred in females and young children below 5 years old (2). Similar to the present study, the increased risk of the rs8506 TT variant genotype was more evident in the children of 12–60 months old and in females, as compared with the CC/CT genotypes. Furthermore, it is surprising in our study, the rs8506 TT genotype was markedly associated with an increased sepsis risk among the early stage of sepsis and survivor subgroup of the patient cohort, but not severe sepsis, septic shock or non-survivor. Owing to the robustly low frequency of rs8506 TT genotype in the enrolled cohort, we considered that the sample size was not enough to test the power of analysis. The current study is only an investigation that focus on the relationship between gene polymorphism and disease susceptibility. Therefore, more mechanistic studies are needed to confirm the roles of *lincRNA-NR_024015* rs8506 TT in the progression of sepsis in children.

Although this is the first study to evaluate the association between *lincRNA-NR_024015* rs8506 polymorphism and sepsis risk in southern Chinese children, several possible limitations should be addressed in present study. First, there are only 474 sepsis patients and 678 controls included. Therefore, the sample size in the current study might have impact on the test power of statistical analysis. Second, only rs8506 T allele was under investigation in the present study, other *lincRNA-NR_024015* gene polymorphisms with potential function remain to be took into consideration. Third, we did not apply other valuable factors that are also important for sepsis severity and prognosis in the present (i.e., scores of sepsis-related organ failure assessment, length of hospital stay) (1). Forth, it has been shown that many factors (i.e., living environment, social-economic factor, population education) have impact on the incidence of sepsis (2, 40); however, we could only collect frequency-matched cases and controls by age and gender due to lack of these information.

## Conclusion

In summary, we verified a significant association between *lincRNA-NR_024015* rs8506 TT genotype and increased sepsis susceptibility in southern Chinese children, especially for children aged 12–60 months, females, and those with early stage of sepsis. Future studies with larger sample size and mechanistic experiments should be conducted to strengthen our findings.

## Data Availability Statement

The raw analyzed datasets used in the study will be available from the corresponding authors on reasonable request.

## Ethics Statement

The studies involving human participants were reviewed and approved by this study had received the Ethics approval of the Institutional Review Board of Guangzhou Women and Children's Medical Center (2015042202). Written informed consent was obtained from each participant guardian. Written informed consent to participate in this study was provided by the participants' legal guardian/next of kin.

## Author Contributions

JL and HZ designed the experiments. JL, HZ, and BW performed the experiments. DC and YX analyzed the data. LP and LF collected the samples and clinical data. JL wrote the manuscript. JH and XG revised and finalized the manuscript. All authors contributed to the article and approved the submitted version.

## Funding

This study was funded by the Guangdong Basic and Applied Basic Research Foundation (grant number 2021B1515230003), the Guangdong Natural Science Fund, China (grant numbers 2019A1515012061, 202102020829, and 2022A1515012558), the Guangzhou Science and Technology Program Project, China (grant numbers 201904010486, 202102010197, 202102021144, and 202102020829), the Subject Construction Project of Guangzhou Medical University (grant number 02-410-2206062), Doctoral Research Initiation Fund from Guangzhou Institute of Pediatrics, Guangzhou Women and Children's Medical Center (grant number 1600104), and Post-doctoral Research Initiation Fund from Guangzhou Institute of Pediatrics, Guangzhou Women and Children's Medical Center (grant numbers 3001162 and 3001178-04).

## Conflict of Interest

The authors declare that the research was conducted in the absence of any commercial or financial relationships that could be construed as a potential conflict of interest.

## Publisher's Note

All claims expressed in this article are solely those of the authors and do not necessarily represent those of their affiliated organizations, or those of the publisher, the editors and the reviewers. Any product that may be evaluated in this article, or claim that may be made by its manufacturer, is not guaranteed or endorsed by the publisher.
